# Factors Influencing the Implementation of School Wellness Policies in the United States, 2009

**DOI:** 10.5888/pcd9.110296

**Published:** 2012-06-28

**Authors:** Elizabeth L. Budd, Cynthia Schwarz, Byron W. Yount, Debra Haire-Joshu

**Affiliations:** Author Affiliations: Cynthia Schwarz, Byron W. Yount, Debra Haire-Joshu, Washington University in St. Louis, Missouri.

## Abstract

**Introduction:**

The quality of school wellness policy implementation varies among schools in the United States. The objective of this study was to characterize the school wellness policy environment nationally and identify factors influencing the quality and effectiveness of policy implementation.

**Methods:**

We invited school administrators from 300 high schools to complete a questionnaire; 112 administrators responded. We performed a 2-step cluster analysis to help identify factors influencing the implementation of school wellness policies.

**Results:**

Eighty-two percent of schools reported making staff aware of policy requirements; 77% established a wellness committee or task force, 73% developed administrative procedures, and 56% trained staff for policy implementation. Most commonly reported challenges to implementation were lack of time or coordination of policy team (37% of respondents) and lack of monetary resources (33%). The core domains least likely to be implemented were communication and promotion (63% of respondents) and evaluation (54%). Cluster 1, represented mostly by schools that have taken action toward implementing policies, had higher implementation and effectiveness ratings than Cluster 2, which was defined by taking fewer actions toward policy implementation. In Cluster 1, accountability was also associated with high ratings of implementation quality and effectiveness.

**Conclusion:**

The development of organizational capacity may be critical to ensuring an environment that promotes high-quality policy implementation. Assessing, preventing, and addressing challenges; establishing clear definitions and goals; and requiring accountability for enacting policy across all core domains are critical to ensuring high-quality implementation.

## Introduction

The Child Nutrition and WIC Reauthorization Act (CNRA) of 2004 required all schools receiving federal funding to have a local school wellness policy (SWP) in place by 2006 (1). Several studies that have assessed the effect of SWPs have found varying results (2-6). The reauthorization in 2010 of the CNRA, also called the Healthy, Hunger-Free Kids Act (HHFKA), established SWP standards in nutrition, access to school meals, and program monitoring (7).

Evidence-based policies are not sufficient in achieving intended outcomes (8). A policy does not end with its development but rather is an initial step in the policy-to-action continuum (9). A supportive environment is critical not only for policy development but also for effective implementation (10-12). Discrepancies exist in the quality of SWP implementation among schools (13,14). Differences in moderating and mediating factors, such as leadership and stakeholder buy-in, adequate resources, and effective feedback and accountability systems, contribute to these discrepancies (10,15). These factors are among those needed to ensure that SWPs are implemented most effectively to meet the intended goals (10,15). Alternatively, the most carefully crafted SWP can fail because of improper implementation in an unsupportive policy environment (11). The objective of this study was to characterize the SWP environment nationally and identify factors influencing the quality and effectiveness of policy implementation.

## Methods

This was a cross-sectional study. We surveyed leaders from a national sample of 300 high schools and addressed the following questions: 1) What actions have schools taken to implement an SWP?, 2)What are the challenges associated with implementation of an SWP?, 3) Are core domains of an SWP being implemented consistently and effectively?, and 4) Does accountability for an SWP impact implementation quality? The institutional review board of Washington University in St. Louis approved this study.

High schools in which students participated in Parents as Teachers, a national parenting and child development program, were selected to participate in Moms for a Healthy Balance (BALANCE) (16-18). BALANCE was a weight-control study of postpartum adolescents in 300 high schools in 27 states; the study is described elsewhere (19). From March through November 2009, we contacted 1 administrator from each of the 300 BALANCE high schools. Administrators were sent a written questionnaire via e-mail, fax, or mail, according to each administrator’s preference. We asked the administrator to deliver the questionnaire to the person responsible for ensuring that the school fulfills the district’s SWP. We applied no additional inclusion or exclusion criteria. We e-mailed each school 3 times; we made a fourth attempt by telephone and a fifth attempt via mail. We made a total of 1,403 attempts: 835 e-mails, 411 telephone calls, 121 faxes, and 36 mailings.

### Measures

The SWP Implementation Questionnaire (SWP-IQ) is a 27-item written survey developed to assess variables influencing SWP implementation (Appendix). We developed the SWP-IQ on the basis of an extensive literature review; it was pilot tested for face validity and relevance with eligible respondents before it was administered in this study. The SWP-IQ was divided into 4 sections. The first section assessed whether the school took action to implement an SWP (5 possible actions; answers of yes, no, or “not sure”). The second section assessed challenges to SWP implementation; 10 challenges were identified, and respondents were asked to check all that apply. The third section asked respondents to evaluate the quality of implementation, or how well (scale of 1–10; 1 = very poor, 10 = excellent) an SWP was implemented across the 7 core domains as recommended by the CNRA: 1) nutrition education, 2) standards for United States Department of Agriculture (USDA) child nutrition programs and school meals, 3) nutrition standards for competitive and other foods and beverages, 4) physical education, 5) physical activity, 6) communication and promotion, and 7) evaluation. This same section also asked respondents to assess how effective (scale of 1–10; 1 = ineffective, 10 = very effective) the policy had been across the 7 core domains (1). The fourth section assessed whether the school was held accountable for implementing each of the core domains (answer of yes or no).

### Data analysis

We evaluated the survey responses and organized them as rank-ordered percentages. We then used a 2-step cluster analysis to examine whether respondent clusters formed after combining attributes on SWP implementation actions and challenges into 1 model. Because 2-step cluster analysis is sensitive to respondent order, we performed 3 consecutive 2-step cluster analyses with random respondent ordering, settling discordance in cluster assignment by selecting the most common assignment for each respondent. The optimal number of clusters was determined by the Schwarz Bayesian Information Criterion (BIC), which indicated 2 clusters for all 3 analyses. The quality of cluster solutions was assessed by using the silhouette coefficient, a measure of both within-cluster cohesion and between-cluster separation. Clusters were then reviewed for differentiating items and compared for similarity of self-reported SWP implementation quality and effectiveness ratings with the Wilcoxon–Mann–Whitney *U* Test. Using χ^2^ tests, we next examined whether being held accountable for implementing policy varied by cluster membership. Last, we assessed whether accountability differentiated SWP implementation quality and effectiveness ratings within clusters.

Statistical significance was predetermined at a 2-tailed α of .05. We screened SWP-IQ data for outliers, distributional anomalies, logical response patterning, variable independence, and normality before analyses. All analyses were performed using Statistical Package for the Social Sciences version 19 (SPSS Inc, Chicago, Illinois).

## Results

Of the 300 high schools contacted, 112 surveys (37.3%) were returned, 179 schools did not respond, 7 schools declined participation, 1 school was unaware of a district wellness policy, and 1 school had closed permanently. Survey responders and nonresponders were similar in the percentage of student population participating in the National School Lunch Program (40%, responders vs 39%, nonresponders; *t*_253_ = 0.36, *P* = .73). Respondents included 44 principals, 23 nurses, 15 food service/nutrition directors, 11 assistant principals, 10 health/wellness coordinators, and 9 others (eg, administrator, athletic director, counselor). Respondents represented 22 states in 4 regions: New York, Pennsylvania, and Rhode Island (Northeast); Illinois, Indiana, Iowa, Kansas, Michigan, Missouri, Ohio, South Dakota, and Wisconsin (Midwest); Alabama, Arkansas, Kentucky, Louisiana, North Carolina, Oklahoma, South Carolina, Tennessee, and Texas (South); and California (West).

### Actions, challenges, quality and effectiveness, and accountability

Most respondents (82%) indicated their school made their staff aware of policy requirements; 77% established a wellness committee or task force, 73% developed administrative procedures to guide policy implementation, and 56% trained staff to implement the policy. Only 26% had acquired funding for implementing the SWP.

The most commonly cited challenges to implementation were lack of time or coordination of policy team (37% of respondents) and lack of monetary resources (33%), followed by “not a priority” (26%), lack of staff cooperation or support (24%), lack of student acceptance (24%), “no consequences of noncompliance” (20%), lack of training, technical assistance, or resources (20%), “lack of knowledge or unsure how to proceed” (17%), lack of leadership (10%), and lack of appropriate food or beverages available from vendors and suppliers (6%).

Five of the 7 policy core domains were reported as being implemented by more than 80% of the schools (nutrition education, nutrition standards for USDA child nutrition programs and school meals, nutrition standards for competitive and other foods and beverages, physical education, and physical activity). The core domains least likely to be implemented were communication and promotion (63% of respondents) and evaluation (54%).

Only 33% of schools were held accountable for implementing communication and promotion; 85% were held accountable for implementing nutrition standards for USDA child nutrition programs and school meals. For 3 domains (physical activity goals, communication and promotion, evaluation), most respondents reported they were either unsure or that there was no accountability for SWP implementation.

### Cluster analysis

Two clusters formed with a silhouette coefficient of 0.30. Cluster 1 had 79 members and was represented mostly by schools that have taken actions toward implementing SWPs (Table 1). Most cluster members reported that their school had developed procedures for implementing SWPs, made staff aware of the SWP, trained staff with access to supportive resources, and designated oversight of the SWP, and appeared sufficiently funded. Cluster 2 had 20 members; the prevalence of aforementioned attributes was low. We were unable to assign 13 schools to either cluster.

**Table 1:** Attributes Associated With School Wellness Policy Implementation, by Order of Importance, National Sample of 112 School Leaders Completing a School Wellness Policy Implementation Questionnaire, 2009^a^

Cluster Attributes	Cluster 1 (n = 79), %			Cluster 2 (n = 20), %		

	Yes	No	Not Sure	Yes	No	Not Sure
Developed administrative procedures to put policy into effect	86	4	10	20	75	5
Made staff aware of policy requirements/developments	95	1	4	30	65	5
Lack of training, technical assistance, or resources available	8	92	0	70	30	0
Trained staff for implementation of the policy	68	15	17	5	90	5
Acquired funding for implementing the policy at your school	33	37	30	0	100	0
Set up wellness policy task force or committee	84	10	6	50	45	5
Lack of monetary resources	27	73	0	60	40	0
Lack of knowledge/not sure how to proceed	13	87	0	35	65	0
Not a priority	23	77	0	40	60	0
Lack of appropriate food/beverages available from vendors and suppliers	8	92	0	0	100	0
Lack of leadership	9	91	0	15	85	0
Lack of time/coordination of policy team	35	65	0	45	55	0
No consequence for noncompliance	22	28	0	15	85	0
Lack of student acceptance	25	75	0	20	80	0
Lack of staff cooperation/support	24	76	0	25	75	0

^a^ Respondents were assigned to clusters using the log-likelihood criterion and the optimal number of clusters determined by the Schwarz Bayesian Information Criterion. Thirteen respondents did not fit well into any cluster and thus were excluded from cluster attribute comparisons.

Cluster 1 was consistently higher than Cluster 2 in ratings for both quality and effectiveness of implementation, although ratings for Cluster 1 varied from 5.5 to 8.0 (Table 2). Both clusters rated communication and promotion and evaluation as their lowest quality and least effective domains.

**Table 2:** Mean School Wellness Policy Implementation Quality and Effectiveness Ratings, by Cluster Membership, a National Sample of 112 School Leaders Completing a School Wellness Policy Implementation Questionnaire, 2009

Core Policy Domain	Mean Quality Rating^a^ (SD)		*P* Value^b^

	Cluster 1	Cluster 2	
Nutrition education	6.8 (1.7)	5.1 (1.6)	.001
Nutrition standards for USDA child nutrition programs and school meals	8.0 (1.8)	6.4 (2.4)	.04
Nutrition standards for competitive and other foods and beverages	7.3 (2.0)	6.4 (1.9)	.09
Physical activity	6.6 (1.8)	5.0 (1.9)	.01
Physical education	7.0 (1.8)	5.5 (2.1)	.008
Communication and promotion	6.1 (1.8)	4.3 (1.4)	.001
Evaluation	5.6 (2.1)	4.4 (1.5)	.01
Overall	6.8 (1.3)	5.3 (1.6)	.004

**Core Policy Domain**	**Mean Effectiveness Rating^c^ (SD)**		***P* Value^b^**

	**Cluster 1**	**Cluster 2**	

Nutrition education	6.3 (1.9)	4.4 (1.5)	.008
Nutrition standards for USDA child nutrition programs and school meals	7.6 (2.1)	5.9 (2.7)	.07
Nutrition standards for competitive and other foods and beverages	7.2 (2.1)	6.1 (1.8)	.02
Physical activity	6.3 (1.9)	4.6 (1.7)	.02
Physical education	6.6 (2.0)	5.2 (2.2)	.07
Communication and promotion	6.0 (2.0)	4.0 (1.4)	.002
Evaluation	5.5 (2.2)	4.1 (1.5)	.004
Overall	6.5 (1.5)	4.9 (1.5)	.001

Abbreviations: SD, standard deviation; USDA, US Department of Agriculture.

^a^ Rated on a scale from 1 (very poor) to 10 (excellent).

^b^ Calculated using Wilcoxon–Mann–Whitney *U* test.

^c^ Rated on a scale from 1 (ineffective) to 10 (very effective).

Accountability for implementing policy core domains was equal in both clusters and therefore was not a differentiating factor in quality and effectiveness. However, accountability was associated with higher SWP implementation quality and effectiveness ratings in Cluster 1 (Table 3).

**Table 3:** Accountability for School Wellness Policy Implementation and Ratings of Implementation Quality and Effectiveness Within Cluster 1, National Sample of 112 School Leaders Completing a School Wellness Policy Implementation Questionnaire, 2009

Core Policy Domain	Mean Quality Rating^a^ (SD)		*P* Value^c^

	Yes^b^	No^b^	
Nutrition education	7.3 (1.8)	6.3 (1.8)	.09
Nutrition standards for USDA child nutrition programs and school meals	7.8 (2.1)	8.0 (1.4)	—^d^
Nutrition standards for competitive and other foods and beverages	7.9 (2.0)	6.4 (1.7)	.05
Physical activity	7.3 (1.7)	5.6 (2.0)	.004
Physical education	7.6 (1.7)	5.3 (2.3)	.006
Communication and promotion	6.6 (1.8)	5.1 (1.8)	<.001
Evaluation	6.9 (1.7)	4.3 (2.2)	.001

**Core Policy Domain**	**Mean Effectiveness Rating^e^ (SD)**		***P* Value^c^**

Nutrition education	7.0 (1.9)	5.4 (1.6)	.02
Nutrition standards for USDA child nutrition programs and school meals	8.3 (1.8)	8.0 (1.4)	—^d^
Nutrition standards for competitive and other foods and beverages	7.9 (1.8)	6.7 (2.4)	.25
Physical activity	7.2 (1.6)	4.9 (2.1)	<.001
Physical education	7.2 (1.8)	4.8 (2.5)	.008
Communication and promotion	7.4 (1.7)	4.4 (2.0)	<.001
Evaluation	6.6 (1.7)	4.0 (1.3)	.001

Abbreviations: SD, standard deviation; USDA, US Department of Agriculture.

^a^ Rated on a scale from 1 (very poor) to 10 (excellent).

^b ^Participants answered the following question with yes or no: “Is your school held accountable for following/implementing the local school wellness policy goals?”

^c^ Calculated using Wilcoxon–Mann–Whitney *U* Test.

^d^ No applicable statistical comparison because of too few responses of “no.”

^e^ Rated on a scale from 1 (ineffective) to 10 (very effective).

## Discussion

Several findings provide insight into factors that characterize a supportive policy environment and their influence on the quality and effectiveness of SWP implementation. First, schools reporting higher SWP quality and effectiveness were more likely to have developed organizational capacity to implement an SWP (eg, developed administrative procedures, made staff aware of SWP requirements, set up a wellness task force or committee). They also reported fewer challenges to implementation than schools reporting lower SWP quality. Our results mirror those reported by other studies identifying factors that interfere with policy implementation, such as the SWP not being considered a priority or the lack of funding or staff training (20-23). HHFKA authorizes additional funds to schools for actions to implement new nutritional standards, technical support, and training for food service providers (7). The additional support authorized by HHFKA appears to be a critical investment in support of action-oriented strategies necessary to ensure successful SWP implementation (8,12,16).

Additionally, SWP implementation is likely related not only to specific challenges but also to the sum effect of multiple challenges. The sheer number of challenges to SWP implementation may represent either a supportive (few challenges) or unsupportive (many challenges) policy environment and influence the dynamics of SWP implementation. Our results suggest that actions to support organizational capacity may be critical in limiting challenges to effective SWP implementation. Further work is needed to systematically assess, prevent, and address challenges in the school environment (12,16).

We also found that many core domains of SWPs were not being implemented consistently and effectively. This finding reflects the findings of others who reported variations in both content and implementation of SWP policies (4,5,13). We found that the domains most likely to be implemented were those that were mandated or were associated with specific criteria (eg, physical activity). In contrast, the domains of evaluation and communication and promotion were least likely to be implemented, perhaps because they are more broadly defined or costly to implement (8). HHFKA authorizes funds for providing information on the school nutrition environment to the community and requires state and federal audits of the implementation of HHFKA requirements. The authorization of funds to support objectives in the communication and promotion and evaluation domains may help ensure the consistent implementation of SWPs. Further work is needed to clarify the domains of evaluation and communication and promotion in a way that is easily definable, measurable, and affordable to schools (7).

Finally, our work further defines the critical role of accountability and its effect on the quality of SWP implementation (3,9,20,24,25). Accountability for SWP can be measured in terms of transparency, oversight, and systematic evaluation (26,27).We found that accountability for SWP implementation varied widely by core domain and that quality of implementation was affected by level of accountability. More specifically, respondents who were held accountable for implementing core SWP domains were significantly more likely to report the presence of positive attributes (eg, many actions taken toward SWP implementation, few challenges) that may be responsible for the higher implementation ratings. In contrast, respondents with less accountability reported overall lower quality implementation. Accountability may moderate the effect of these positive attributes or have an independent effect on implementation ratings. Future studies should more closely examine the role of accountability in the implementation of quality SWP.

HHFKA encourages SWP accountability by requiring regular district audits of nutritional compliance. More enhanced accountability is needed to support SWP actions associated with the other core domains (eg, physical education, physical activity, evaluation). Requiring accountability across all SWP core domains could be a strategy to facilitate optimal policy implementation.

This study had several limitations, which are consistent with cross-sectional study designs and self-report measures. We relied on administrators to ensure the individuals who filled out the SWP-IQ were indeed those responsible for implementing the SWP at their schools. The survey response rate was low, and we were unable to collect information on most of the nonrespondents. We are unable to comment on the generalizability of this study because we have limited information on the school districts who participated.

This study suggests that several characteristics of a policy environment are associated with the quality of SWP implementation. Enhanced organizational capacity may reduce the total number of challenges that affect SWP implementation. Steps to better assess and address challenges in the policy environment are needed to facilitate improvement in the quality of SWP implementation. Additionally, core domains of SWP appear to be implemented selectively and to varying degrees. Clear definitions and goals for all SWP domains are needed to facilitate comprehensive and high-quality implementation of the policy. Finally, accountability for enacting the SWP across all core domains could be critical to ensuring high-quality SWP implementation.

## Appendix. Text of School Wellness Policy Implementation Questionnaire

1. The following are actions that your high school may or may not have taken toward implementation of a local wellness policy. For each, please indicate if this is an action that your high school has taken. (Mark the appropriate box for each item.) [For each item, response is yes, no, or “not sure.”]
  - Set up wellness policy task force or assigned a committee
  - Developed administrative procedures to put policy into effect
  - Made staff aware of policy requirements/developments
  - Trained staff for implementation of the policy
  - Acquired funding for implementing the policy at your school
2. Have there been any challenges with implementation of this policy at your high school that you are aware of? (Choose all that apply.)
  - Lack of monetary resources
  - Lake of training/technical assistance/resources available
  - Lack of knowledge/not sure how to proceed
  - Not a priority
  - Lack of staff cooperation/support
  - Lack of time/coordination of policy team
  - Lack of leadership
  - Lack of appropriate food/beverages available from vendors and suppliers
  - Lack of student acceptance
  - No consequence for noncompliance
3. On a scale of 1–10 with 10 being excellent, how well do you think the following policy goals have been implemented at your school? [Response also includes “Not applicable.”]
  - Nutrition Education Goals
  - Nutrition Standards for USDA Child Nutrition Programs and School Meals
  - Nutrition Standards for Competitive and Other Foods and Beverages
  - Physical Activity Goals
  - Physical Education Goals
  - Communication and Promotion Goals
  - Evaluation Goals
4. On a scale of 1–10 with ten being very effective, how effective do you think this policy has been regarding . . . [Response also includes “Not applicable.”]
  - Nutrition Education Goals
  - Nutrition Standards for USDA Child Nutrition Programs and School Meals
  - Nutrition Standards for Competitive and Other Foods and Beverages
  - Physical Activity Goals
  - Physical Education Goals
  - Communication and Promotion Goals
  - Evaluation Goals
5. Is your school held accountable for following/implementing the local school wellness policy goals? (Mark the appropriate box for each item.) [Possible answer is yes or no.]
  - Nutrition Education Goals
  - Nutrition Standards for USDA Child Nutrition Programs and School Meals
  - Nutrition Standards for Competitive and Other Foods and Beverages
  - Physical Activity Goals
  - Physical Education Goals
  - Communication and Promotion Goals
  - Evaluation Goals
